# The role of gut microbiota in autoimmune thyroid diseases: nutritional determinants and diet-based modulation

**DOI:** 10.3389/fendo.2026.1785878

**Published:** 2026-02-19

**Authors:** Hubert Lewandowski, Maciej Maslyk, Halla Kaminska, Lukasz Szarpak

**Affiliations:** 1 Department of Clinical Research and Development, LUXMED Group, Warsaw, Poland; 2 Institute of Biological Sciences, The John Paul II Catholic University of Lublin, Lublin, Poland; 3 Department of Children’s Diabetology and Lifestyle Medicine, Faculty of Medical Sciences, Katowice, Medical University of Silesia, Katowice, Poland; 4 Institute of Medical Science, The John Paul II Catholic University of Lublin, Lublin, Poland; 5 Henry JN Taub Department of Emergency Medicine, Baylor College of Medicine, Houston, TX,, United States

**Keywords:** autoimmune thyroid diseases, Graves’ disease, gut microbiota, Hashimoto’s thyroiditis, immune modulation, intestinal permeability, nutrition

## Abstract

Autoimmune thyroid diseases (AITD), primarily Hashimoto’s thyroiditis and Graves’ disease, represent the most common organ-specific autoimmune disorders and remain a significant clinical challenge due to their chronic course, frequent comorbidities, and limited options for causal treatment. In recent years, increasing attention has been directed toward the gut microbiota as a potential modulator of immune tolerance and endocrine autoimmunity. Accumulating evidence suggests that alterations in gut microbial composition and function may contribute to immune dysregulation, intestinal barrier dysfunction, and low-grade inflammation observed in patients with AITD. This narrative review summarizes current knowledge on the role of gut microbiota in the pathophysiology of autoimmune thyroid diseases, with a particular focus on nutritional determinants and diet-based strategies for microbiota modulation. We discuss mechanisms linking diet, microbial metabolites, intestinal permeability, and immune responses relevant to thyroid autoimmunity. Special attention is given to dietary patterns, specific nutrients, and bioactive food components that may influence gut microbiota composition and function, including fiber, selenium, iodine, vitamin D, polyphenols, and probiotic-containing foods. From a clinical standpoint, the available evidence remains limited and largely heterogeneous, with most data derived from observational studies rather than interventional trials. Although growing interest surrounds diet-driven modulation of the gut microbiota, current findings do not support its use as an independent therapeutic approach in autoimmune thyroid diseases. Instead, dietary interventions may be best viewed as a complementary element of overall patient care. Well-designed prospective studies are still needed to determine whether such strategies can meaningfully influence disease course, to define which patients may benefit, and to translate these observations into practical, evidence-based dietary guidance. Future progress will depend on function-focused, phenotype-informed studies integrating microbiota readouts with clinically meaningful endocrine endpoints.

## 1 Introduction

Autoimmune thyroid diseases (AITD) primarily include Hashimoto’s thyroiditis (HT) and Graves’ disease (GD). They are among the most common autoimmune disorders, with a prevalence reaching up to 5% in the general population, and are associated with substantial clinical and economic burden (1, 2). HT remains the leading cause of acquired hypothyroidism, whereas GD is the predominant autoimmune cause of hyperthyroidism (1, 2).

The development of autoimmune thyroid diseases is widely recognized as multifactorial, arising from the interplay between genetic predisposition and environmental influences that shape immune responses over time (1, 3, 4). Evidence from twin studies suggests that genetic factors account for a substantial proportion of disease susceptibility, while non-genetic exposures remain critical in determining whether and how autoimmunity manifests clinically (2, 4). Consistent associations have been reported for several immune-related genetic regions, particularly within the HLA locus, supporting the concept that AITD reflects dysregulation of immune tolerance rather than a single, disease-specific genetic defect (1, 3, 4). Additional susceptibility is conferred by genes encoding thyroid-specific antigens, including thyroglobulin and the TSH receptor (1–3, 5).

Among environmental factors, particular attention has been given to nutritional exposures, including iodine, selenium, zinc, iron, and vitamin D status, as well as infections such as Yersinia enterocolitica, cigarette smoking, and the use of selected medications. These include immune checkpoint inhibitors, amiodarone, lithium, and interferons. Chronic psychological stress and periods of physiological immune modulation, such as pregnancy and the postpartum state, also contribute to disease risk (1, 3, 4). Increasing evidence suggests that several of these factors may act indirectly through alterations in the composition and function of the gut microbiota and its metabolites, forming the basis of the gut–thyroid axis concept (6–8).

Thyroid hormones play an important role in gastrointestinal physiology, influencing intestinal motility, secretory activity, and epithelial homeostasis. Through these effects, thyroid function also shapes the intestinal environment in which the gut microbiota develops. Conversely, accumulating evidence indicates that the gut microbiota can modulate thyroid hormone physiology by affecting micronutrient availability, intestinal metabolic processes, and mechanisms involved in enterohepatic hormone circulation (6–9).

From a clinical perspective, the gut microbiota is best understood as a dynamic ecosystem rather than a static trait. In clinical practice, the gut microbiota is influenced by a range of everyday factors, most notably dietary habits, medication use—particularly antibiotics—age, comorbid conditions, and broader environmental exposures. When this balance is disturbed—a state often described as dysbiosis—it may be accompanied by persistent low-grade inflammation, weakening of the intestinal barrier, and broader disturbances in immune regulation (6, 9–11).

Over the past decade, multiple observational and translational studies have compared gut microbiota profiles in patients with Hashimoto’s thyroiditis or Graves’ disease with those of healthy individuals. While the results are not fully consistent, they collectively support a potential link between dysbiosis and impaired immune tolerance to thyroid antigens (6, 11–15). Meta-analyses suggest that patients with Hashimoto’s thyroiditis more often display preserved or increased microbial diversity, whereas Graves’ disease is generally associated with reduced diversity across several metrics (12). Associations between gut microbiota composition and thyroid autoimmunity appear strongest for anti–thyroid peroxidase antibody titers, highlighting a clinically relevant relationship between microbial alterations and autoimmune activity (12).

These findings should be interpreted with caution. Available results remain partly inconsistent, reflecting heterogeneity in study populations, differences in diet and geographic exposure, and substantial methodological variability. Key sources of divergence include the use of 16S rRNA sequencing versus shotgun metagenomics, differences in bioinformatic pipelines, and variable control of confounding factors (6, 7, 12).

The aims of this review are fourfold: (1) to provide a concise synthesis of current evidence on gut microbiota alterations in HT and GD; (2) to discuss mechanisms linking the gut microbiota to thyroid autoimmunity, including intestinal barrier dysfunction, immune modulation, molecular mimicry, and microbiota-derived metabolites; (3) to describe the bidirectional gut–thyroid axis with particular emphasis on nutritional factors and micronutrients; and (4) to critically appraise available dietary and microbiota-targeted interventions that may influence the course of AITD (6, 7, 10, 16–20).

## 2 Materials and methods

### 2.1 Study design

This work is a state-of-the-art narrative review with a targeted and structured literature selection strategy, chosen to address the mechanistic complexity and methodological heterogeneity of gut–thyroid axis research. A formal systematic review was not pursued because of the substantial variability in study design, populations, microbiota assessment methods, and outcome measures, which currently precludes meaningful quantitative synthesis. This approach was chosen due to the complexity of the gut–thyroid axis, which encompasses observational, translational (including animal models), interventional studies, and meta-analyses, alongside substantial methodological and population heterogeneity in the available evidence (8, 21). The aim was not to conduct a formal systematic review, but to provide a critical synthesis of current knowledge from both clinical and mechanistic perspectives. This review was conducted as a narrative, state-of-the-art synthesis; therefore, formal systematic review frameworks such as PRISMA were not applied.

### 2.2 Literature search strategy and selection

Key publications were identified through searches of PubMed/MEDLINE, Web of Science, and Scopus. The literature search covered publications from January 2009 through December 2025. The search strategy was complemented by manual reference screening (snowballing) of systematic reviews, meta-analyses, and landmark original studies. Combinations of keywords and MeSH terms were used, including “Hashimoto,” “Graves,” “autoimmune thyroid disease,” “gut microbiota/microbiome,” “dysbiosis,” “intestinal permeability,” “short-chain fatty acids,” “probiotics,” “prebiotics,” “synbiotics,” “diet,” “iodine,” “selenium,” and “vitamin D.” Eligible studies addressed gut microbiota composition and function and their metabolites in HT and/or GD, as well as investigations evaluating the effects of dietary and microbiota-targeted interventions on clinical and immunological outcomes. To reduce selection bias, the search strategy prioritized systematic reviews, meta-analyses, and controlled studies where available, and findings were interpreted with explicit consideration of study design, population characteristics, and potential confounders.

### 2.3 Inclusion and exclusion criteria

Preference was given to publications meeting at least one of the following criteria: (1) studies with a control group, including case–control and cohort designs; (2) analyses examining associations between gut microbiota composition and markers of thyroid autoimmunity (anti-TPO, anti-Tg, TRAb antibodies); (3) randomized interventional trials involving dietary modification, probiotics, prebiotics, synbiotics, or micronutrient supplementation; and (4) systematic reviews and meta-analyses.

Studies of a purely theoretical nature, reports lacking clearly described microbiota analysis methods, and publications in which the effects of AITD could not be disentangled from major confounders—such as recent antibiotic exposure without adequate control—were excluded.

### 2.4 Evidence synthesis

The collected data were organized according to the IMRAD framework. In the Results section, findings are presented by disease entity (HT versus GD), key mechanistic pathways (intestinal barrier function, immunomodulation, microbiota-derived metabolites, molecular mimicry), and type of intervention (dietary approaches, probiotics/prebiotics/synbiotics, supplementation, and other therapeutic strategies). The Discussion provides a critical appraisal of result consistency and study limitations, with particular attention to the impact of dietary variability, geographic factors, and methodological differences across studies (8, 21, 22). Where inconsistencies between studies were identified, emphasis was placed on convergent functional pathways rather than isolated taxonomic findings, in order to minimize overinterpretation of context-dependent results.

## 3 Results

### 3.1 Gut microbiota alterations in Hashimoto’s thyroiditis

In Hashimoto’s thyroiditis (HT), gut microbiota dysbiosis has been consistently reported. The pattern of alterations remains heterogeneous and depends on the studied population, environmental context, and dietary exposures. Meta-analyses and systematic reviews describe disturbances in both alpha diversity and the relative abundance of selected taxa at the genus and species levels, while emphasizing substantial between-study heterogeneity and possible geographic clustering of findings (12, 23).

In a Brazilian cohort, patients with HT showed an increased abundance of Bacteroides and a reduced abundance of Bifidobacterium. Where reported at higher resolution, changes frequently involve genera linked to carbohydrate fermentation and mucosal immune tone (e.g., Bifidobacterium, Lactobacillus, Bacteroides), although the direction of change varies across cohorts. Mechanistically, these shifts are most plausibly interpreted through functional consequences—SCFA output, epithelial integrity, and downstream antigen exposure—rather than a single disease-specific taxonomic signature. Higher circulating zonulin levels were reported alongside microbiota alterations, consistent with barrier perturbation; however, zonulin is an indirect, assay-dependent signal and should be interpreted cautiously (see Limitations of the Evidence) (15, 24–26). Similar observations have been made in other cohorts, although the specific microbial changes differ from one study to another (27). Notably, despite this variability, the reported alterations tend to converge at the level of microbial function rather than individual taxa. This observation reinforces the view that reported taxonomic differences in Hashimoto’s thyroiditis are more likely to reflect underlying functional disturbances of the gut microbiota rather than a single, disease-specific microbial signature.

Against this background, increasing attention has focused on iodine as a potential modulator of both gut microbiota composition and immune responses. Translational studies indicate that excessive iodine intake may disrupt metabolic pathways related to butyrate and propionate metabolism and promote a shift toward a pro-inflammatory immune phenotype that favors thyroid autoimmunity (27, 28). These effects include impaired SCFA synthesis and altered redox balance, providing a functional link between iodine exposure, gut dysbiosis, and immune dysregulation.

Associations have also been reported between specific microbial taxa and the severity of thyroid autoimmunity, as reflected by anti-TPO and anti-Tg antibody titers (12, 23, 28). Interpretation of these relationships is complicated by disease stage, thyroid hormone replacement, and lifestyle modifications. Diet remains one of the main determinants of gut microbiota composition and should be systematically assessed in microbiota studies in HT (15, 23).

### 3.2 Gut microbiota alterations in Graves’ disease and Graves’ orbitopathy

In Graves’ disease (GD) and its extrathyroidal manifestation, Graves’ orbitopathy (GO), a marked reduction in gut microbiota diversity is commonly observed. This is typically accompanied by an increased abundance of Bacteroidota and a relative depletion of Firmicutes. These changes correlate with the severity of hyperthyroidism and with higher titers of TSH receptor antibodies (TRAb) (29, 30). In parallel, several studies implicate a pro-inflammatory immune phenotype in active disease, including increased Th17-associated signaling (e.g., IL-17/IL-21) and reduced regulatory tone. This pattern is consistent with enhanced antigen presentation by dendritic cells and macrophage activation in the context of barrier perturbation and altered microbial metabolite profiles.

In patients with Graves’ orbitopathy, several studies have reported gut microbiota changes that tend to track with disease activity and the intensity of the autoimmune response (31–33). Clinically, these findings fit with a broader pattern that includes impaired barrier function, systemic inflammatory features, and progression of orbital involvement (30, 31). From a functional perspective, the available data point toward altered microbial metabolism, which may favor immune profiles associated with disease activity rather than immune regulation.

With effective antithyroid treatment, changes in the gut microbiota appear to move in a more physiological direction, a pattern that tends to coincide with clinical and biochemical improvement. These changes parallel clinical improvement and reduced autoimmune activity (29). In animal models and translational studies, fecal microbiota transplantation from patients with GO attenuated disease manifestations, whereas microbiota modulation, including antibiotics or probiotics, attenuated disease manifestations (34, 35). However, these experimental findings require confirmation in adequately powered human interventional trials.

Probiotic supplementation, particularly with *Faecalibacterium prausnitzii*, and butyrate administration show therapeutic potential by inhibiting orbital fibroblast activation, reducing fibrosis and adipogenesis, and lowering TRAb levels (36, 37). At the same time, some animal models suggest that specific probiotic strains may exacerbate autoimmunity, underscoring the need for cautious interpretation and further clinical validation (35).

From a clinical perspective, smoking is consistently associated with a more severe course of Graves’ disease and Graves’ orbitopathy, although the underlying mechanisms are likely multifactorial (29, 38). Clinical observations further suggest that managing chronic oral infections may be accompanied by improvement in orbitopathy, pointing to a broader role of mucosal microbial factors in GD/GO (38).

Taken together, dysbiosis in GD and GO is closely linked to disease activity, TRAb titers, clinical course, and treatment response. Environmental modification, including smoking cessation, management of oral infections, and targeted microbiota interventions, may represent valuable adjuncts to conventional therapy (29–33, 35–38).

**Figure 1:**
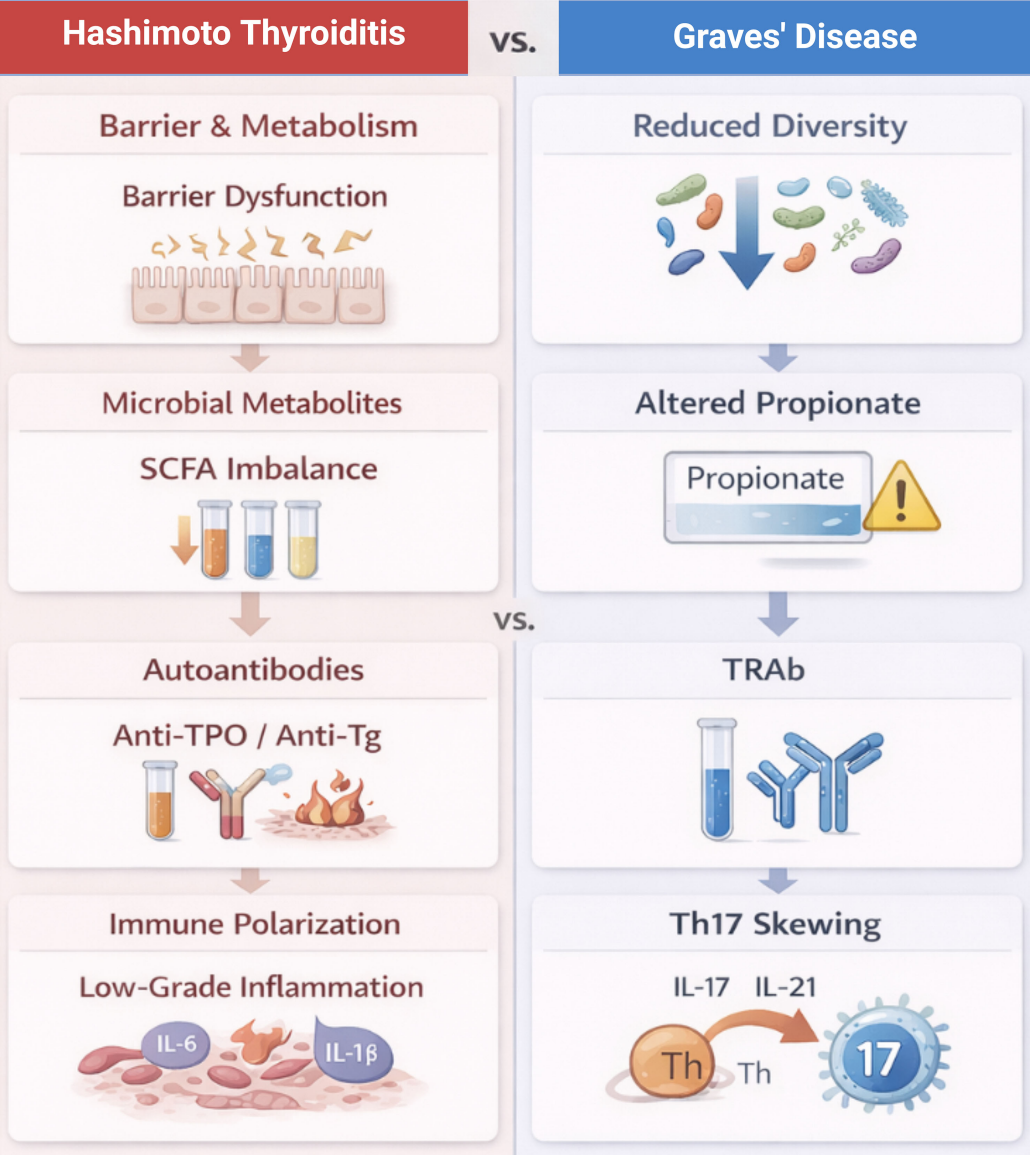
Microbial functional pathways linking environmental factors, immunity, and autoimmune thyroid disease.

Taken together, the available evidence suggests that Hashimoto’s thyroiditis and Graves’ disease differ less in specific microbial taxa and more in the way gut microbial function is altered. In Hashimoto’s thyroiditis, these changes tend to cluster around impaired barrier function, disturbances in microbial metabolism, and a background of persistent low-grade inflammation. GD is characterized by reduced microbial diversity, impaired propionate metabolism, and Th17-skewed immune responses. These functional contrasts between HT and GD are summarized in **Figure 1**.

For clarity, the main differences between Hashimoto’s thyroiditis and Graves’ disease are brought together in **Table 1**. Rather than listing individual taxa, the table highlights recurring functional features of microbiota alterations that are more relevant from a clinical perspective.

**Table 1:** Functional patterns of gut microbiota alterations in autoimmune thyroid diseases.

Functional domain	Hashimoto’s thyroiditis (HT)	Graves’ disease (GD/GO)	Clinical/mechanistic relevance
Microbial diversity	Preserved or increased; highly heterogeneous across studies	Reduced diversity, particularly in active disease	Reflects distinct disease phenotypes and context-dependent immune activation patterns
Intestinal barrier function	Barrier dysfunction; higher zonulin (assay-dependent) and other indirect permeability signals*	Barrier impairment, especially in active GD/GO; higher LPS/LBP, D-lactate, I-FABP and related markers; zonulin reported (assay-dependent)*	Facilitates antigen translocation and systemic immune activation
Short-chain fatty acids (SCFA)	Imbalance in butyrate- and propionate-related microbial capacity	Altered SCFA profile with reduced propionate-related metabolic signaling	Modulates Th17/Treg balance and mucosal immune regulation; supports epithelial integrity
Immune response profile	Chronic low-grade inflammation	Th17-skewed immune response	Associated with disease-specific immune activation patterns and activity
Autoantibodies	Anti-TPO/anti-Tg	TRAb	Correlates with disease activity and clinical phenotype
Key modifying factors	Dietary patterns, iodine exposure, micronutrient status	Smoking, orbitopathy severity/activity, antithyroid drug therapy	Potential targets for pragmatic adjunctive strategies and key confounders in microbiome studies

*Zonulin measurements are assay-dependent; several commercial ELISA kits may not specifically quantify pre-haptoglobin 2 and should be interpreted as indirect, non-specific barrier-related signals.

### 3.3 Pathogenetic mechanisms linking the gut microbiota to thyroid autoimmunity

**Figure 2:**
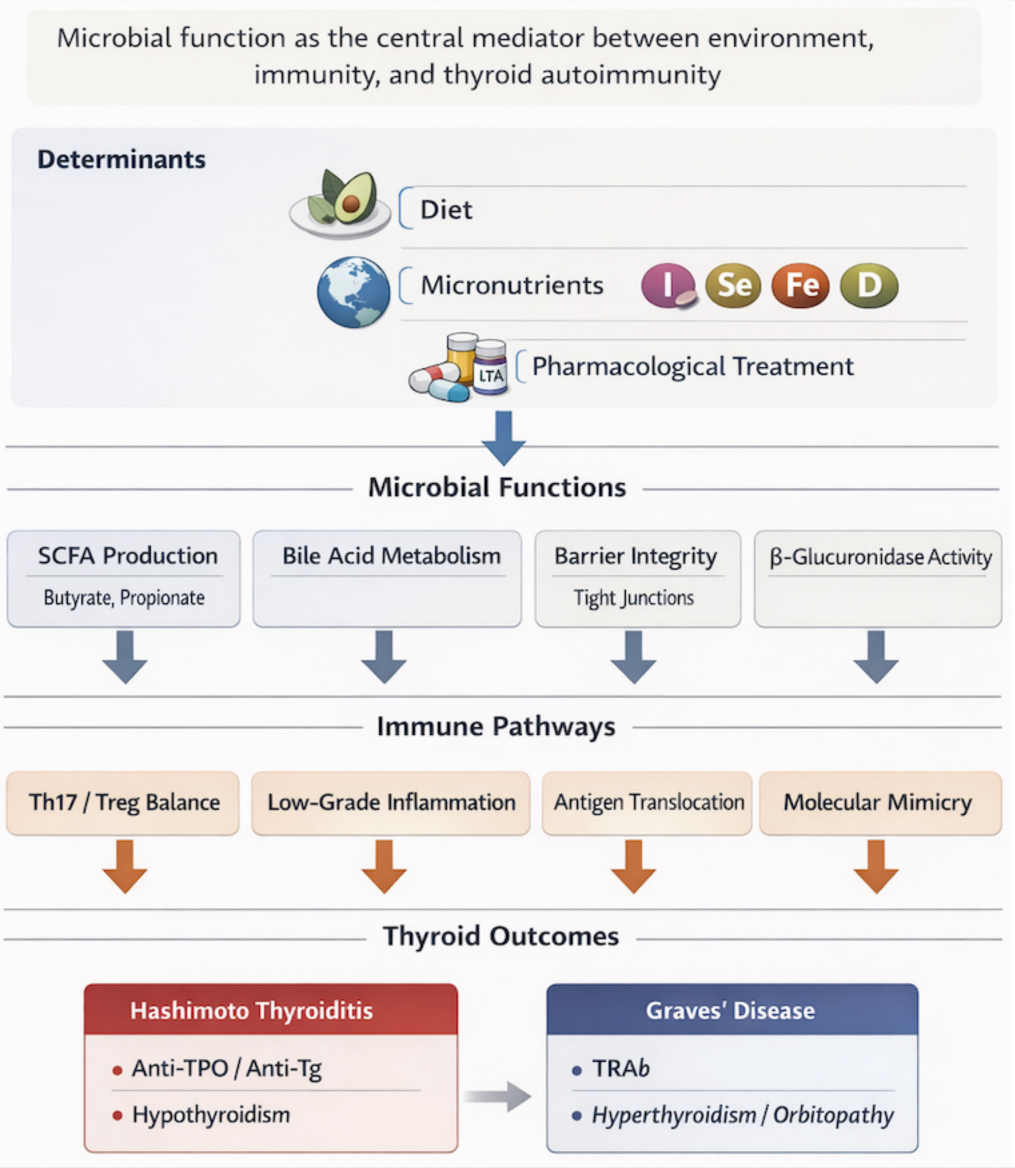
Key functional patterns linking the gut microbiota to Hashimoto’s thyroiditis (HT) and Graves’ disease (GD).

The link between the gut microbiota and thyroid autoimmunity is unlikely to be explained by taxonomic shifts alone. More plausibly, it reflects clinically meaningful functional derangements at the mucosal interface—where environmental exposures are translated into immune dysregulation with downstream endocrine effects. The most consistently implicated mechanisms include altered microbial metabolite output, compromised intestinal barrier function, and immune modulation; acting in concert, these processes may facilitate both the initiation and perpetuation of Hashimoto’s thyroiditis (HT) and Graves’ disease (GD) (**Figure 2**).

As outlined in **Figure 2**, diet, micronutrient status, geography, and commonly used pharmacotherapies can reshape key microbial functions, notably short-chain fatty acid (SCFA) production, bile acid metabolism, and epithelial integrity. These functional changes have direct relevance to established immunopathways—shifting the Th17/Treg balance, increasing antigen translocation across a “leaky” barrier, and potentially promoting molecular mimicry—thereby creating conditions that favor the development and persistence of thyroid autoimmunity (6, 10, 11).

Dysbiosis has been linked to AITD through several interconnected mechanistic axes: (1) disruption of the intestinal barrier, (2) immune modulation via the Th17/Treg axis, (3) molecular mimicry between microbial and thyroid antigens, and (4) actions of microbiota-derived metabolites—particularly SCFA and secondary bile acids—acting through G protein–coupled receptors and epigenetic mechanisms (6, 10, 11). Across these axes, a common mechanistic ‘bottleneck’ is mucosal antigen sensing (e.g., LPS–TLR4 signaling), shaping antigen presentation and T-cell polarization (Treg/Th17) with downstream B-cell activation and autoantibody generation.

#### 3.3.1 Intestinal barrier dysfunction and antigen translocation

Heightened intestinal permeability (“leaky gut”) may increase immune exposure to luminal triggers—most notably bacterial products such as lipopolysaccharide (LPS) as well as incompletely processed dietary antigens—and can therefore plausibly amplify autoimmune responses. In Hashimoto’s thyroiditis, elevated circulating zonulin has been reported as a proxy of barrier perturbation (15). In Graves’ disease, higher levels of LPS, D-lactate, and intestinal fatty acid–binding protein (I-FABP)—markers reflecting endotoxemia and enterocyte injury—have been described and appear to track with TRAb titers and the biochemical/clinical severity of thyrotoxicosis (39). Because circulating zonulin is an indirect, assay-dependent signal, barrier-related claims are best supported by triangulation using complementary markers (e.g., I-FABP; LPS or LBP/sCD14; D-lactate) and, when feasible, functional permeability testing such as urinary sugar probe assays (e.g., lactulose/mannitol ratio) (see also Limitations of the Evidence) (24–26, 40). From a mechanistic perspective, dysbiosis may contribute to tight-junction disruption, activation of innate inflammatory signaling, and erosion of mucosal tolerance. At the molecular level, barrier disruption is commonly framed in terms of altered tight-junction architecture (e.g., occludin/claudins/ZO-1) and enhanced translocation of microbial ligands. These ligands can amplify innate inflammatory pathways (e.g., LPS–TLR4–NF-κB), promoting cytokine release and facilitating a feed-forward loop that sustains systemic, low-grade inflammation. Overall, this pattern favors microbial translocation and a low-grade systemic inflammatory response—conditions that may help sustain thyroid-specific autoimmunity over time (9, 11). This mechanism is clinically relevant given the frequent coexistence of AITD with autoimmune gastrointestinal disorders, such as celiac disease (8).

#### 3.3.2 Immunometabolism: SCFA, bile acids, and the Th17/Treg axis

Short-chain fatty acids (SCFAs) - mainly butyrate, propionate, and acetate—are produced when gut bacteria ferment dietary fiber. They are increasingly viewed as clinically relevant immunometabolic signals, in part because they can shift the balance between Th17-driven inflammation and regulatory T-cell activity (6, 41, 42). In Graves’ disease, dysbiosis has been linked to lower propionate production, which tends to go hand in hand with a more pro-inflammatory T-cell profile and less effective regulatory control (42). Against this background, a propionate-producing strain, Bacteroides fragilis YCH46, has been associated with an increase in regulatory T cells and a reduction in Th17-type responses. Clinically, this is of interest because it suggests a pathway that might be amenable to intervention, although the evidence remains preliminary (42). More broadly, taxa enriched for carbohydrate fermentation and bile-acid transformation sit at the interface of diet, mucosal barrier function, and immune homeostasis, providing a functional bridge between dietary exposures and immune programs relevant to thyroid autoimmunity.

SCFAs appear to mediate their immunoregulatory effects through several converging mechanisms, including inhibition of histone deacetylases, signaling via GPR41, GPR43, and GPR109A, and shifts in T-cell metabolism. Collectively, these pathways favor regulatory T-cell differentiation and augment interleukin-10 production (11, 41, 43, 44). Among the major SCFAs, butyrate is most consistently associated with the most pronounced immunomodulatory effects and, importantly from a clinical standpoint, also supports intestinal barrier integrity and elements of innate immune defense (11). Secondary bile acids—formed through bacterial conversion of primary bile acids—have clinically relevant immunomodulatory and metabolic effects. They may also intersect with thyroid physiology by influencing enterohepatic handling of thyroid hormones, the bioavailability of antithyroid medications, and local iodothyronine metabolism (7, 10, 41, 45). Mechanistically, secondary bile acids signal through receptors such as FXR and TGR5, which can modulate inflammatory tone and immune-cell activity, including pathways relevant to Treg/Th17 balance. This makes bile-acid metabolism a plausible ‘functional node’ linking microbial ecology with immune regulation in AITD. Beyond direct T-cell effects, SCFAs can condition antigen-presenting cells (e.g., dendritic cells) toward tolerogenic phenotypes and support epithelial programs relevant to mucosal tolerance (mucus layer, antimicrobial peptides, IgA-associated homeostasis). This provides a mechanistic bridge between dietary fiber availability, microbial fermentation capacity, and the immune balance that is repeatedly implicated in AITD.

In addition to SCFAs and bile acids, other microbiota-related metabolites may be relevant, including tryptophan derivatives such as indole compounds (e.g., indole-3-propionic acid, indole-3-acetic acid) and metabolites from the kynurenine pathway. These molecules can influence epithelial barrier function and immune signaling (e.g., via AhR-mediated pathways) and therefore may contribute to inflammation-related mechanisms in autoimmune thyroid diseases (6, 46). Notably, indole–AhR signaling has been linked to epithelial barrier support and mucosal immune calibration, providing another mechanistic route by which microbial metabolism may shape inflammatory propensity.

#### 3.3.3 Molecular mimicry and loss of immune tolerance

The molecular mimicry hypothesis argues that immune cross-reactivity can arise when microbial proteins share structural features with key thyroid antigens—most commonly thyroid peroxidase, thyroglobulin, or the TSH receptor—thereby lowering the threshold for activation of autoreactive T cells and sustaining autoimmune responses (11, 47, 48). Sequence homology has been reported between thyroid antigens and proteins derived from several organisms, including Lactobacillus, Bifidobacterium, Yersinia enterocolitica, Helicobacter pylori, and Borrelia burgdorferi; importantly, some of these shared regions overlap with epitopes known to be recognized by human T cells (48–50). A plausible immunological route is that microbial epitopes are processed by antigen-presenting cells (e.g., dendritic cells) and presented in a pro-inflammatory context, lowering the activation threshold for autoreactive T cells. This can facilitate T-cell help to B cells (including Tfh–B cell interactions), thereby strengthening the autoantibody response (anti-TPO/anti-Tg or TRAb depending on phenotype). That said, while the concept is biologically credible and supported by experimental observations, the clinical signal remains largely circumstantial. At present, molecular mimicry should be viewed as a plausible contributor rather than an established driver, and it will require confirmation in well-designed functional studies and prospective cohorts that can link specific exposures to immune readouts and clinically meaningful thyroid outcomes (47, 48, 51).

### 3.4 The bidirectional gut–thyroid axis and thyroid hormone metabolism

The thyroid gland and the gastrointestinal tract are closely linked through bidirectional physiological interactions (6, 8, 10). Hyperthyroidism tends to speed up intestinal transit, whereas hypothyroidism slows it. Those motility changes can alter the intraluminal milieu and, in turn, the microbiota profile—one reason cross-sectional associations are difficult to interpret and causality is hard to establish (7, 45). The microbiota may also matter from a pragmatic therapeutic perspective. By shaping enterohepatic handling of hormones and drugs—partly through bacterial β-glucuronidase and sulfatase activity—and by influencing barrier function, bile-acid dynamics, and micronutrient trafficking, it can plausibly affect levothyroxine absorption and overall drug bioavailability (7, 8, 10, 45). In routine practice, it is often the “usual suspects” that have the clearest impact: comorbid conditions such as coeliac disease or Helicobacter pylori infection can impair levothyroxine absorption, and effective treatment has been associated with a clinically meaningful reduction in levothyroxine dose requirement (reported as up to ~34% in some series) (52). Microbiota composition also correlates with the levothyroxine dose needed to maintain stable TSH levels (53).

### 3.5 Gut microbiota and micronutrients essential for thyroid function

In AITD, iodine, selenium, iron, zinc, vitamin D, and vitamin B12 are of particular importance for thyroid hormone synthesis and metabolism as well as immune regulation (8, 19, 20, 54–56).

Both iodine deficiency and excess may promote thyroid autoimmunity. Translational data indicate that high iodine intake alters gut microbiota composition and shifts immune responses toward a pro-inflammatory phenotype (6, 54–56). Selenoproteins are critical for thyroid hormone metabolism and antioxidant defense. Selenium supplementation has been linked to lower anti-TPO antibody titers in some cohorts; however, whether this biochemical change results in tangible clinical benefit—such as improved symptoms, reduced levothyroxine requirement, or better long-term thyroid function—remains uncertain (8, 20, 54, 55).

Lower serum 25(OH)D levels are more frequently observed in patients with AITD, but interpretation is limited by multiple confounders (8, 19, 20, 54, 55). Zinc and iron deficiencies, particularly in patients with malabsorption, may impair thyroid function. Iron is a cofactor for thyroid peroxidase (TPO), and iron deficiency can therefore be a clinically relevant contributor to suboptimal thyroid hormone synthesis. In practice, correcting iron deficiency may also improve the response to levothyroxine in selected patients, particularly when persistent symptoms or unexpectedly high dose requirements suggest impaired treatment effectiveness (8, 19, 20, 54–56).

### 3.6 Therapeutic interventions targeting the gut microbiota in AITD

#### 3.6.1 Diet as a primary modulator of the microbiota

Diet is one of the most potent and rapidly acting modulators of gut microbiota composition and function (20, 54, 55). In AITD, dietary fiber intake, fat quality, degree of food processing, and consumption of fermented foods are particularly relevant (20, 54). A Mediterranean-style diet—built around vegetables, fruit, legumes, whole grains, nuts, and olive oil, with regular but not excessive fish intake—has been associated with a more favorable gut milieu and a less pro-inflammatory immune profile, alongside improved oxidative balance (54). A key component is dietary fiber, which gut bacteria ferment into short-chain fatty acids; these metabolites are linked to lower inflammatory signaling and better maintenance of the intestinal barrier (20, 55). From a practical standpoint, dietary measures seem to influence what the microbiota does (metabolic output and barrier effects) more than which individual organisms are present, and that functional shift may be the more clinically relevant signal than taxonomy alone.

#### 3.6.2 Probiotics, prebiotics, and synbiotics

Key functional patterns linking the gut microbiota to Hashimoto’s thyroiditis (HT) and Graves’ disease (GD).Across meta-analyses, probiotics, prebiotics, and synbiotics have shown at best modest—and not entirely consistent—signals in thyroid disease, with most outcomes clustering around small changes in inflammatory indices rather than clear endocrine effects (10, 19, 56). In aggregate, supplementation has not produced meaningful shifts in TSH, fT4, or fT3, although a small reduction in TRAb titers has been reported in Graves’ disease in some analyses (19). In Hashimoto’s thyroiditis, a program combining Lactiplantibacillus plantarum 299v with nutritional counselling improved patient-reported quality of life, but anti-TPO titers remained unchanged (6). Across the available studies, differences in probiotic strains, dosing, and duration of treatment make the evidence difficult to generalize, and at present do not support firm, practice-changing recommendations (10, 19, 56, 57). Accordingly, any clinical use should be framed as adjunctive and symptom-oriented.

Evidence supporting probiotics in AITD remains limited and heterogeneous and does not justify disease-modifying recommendations. If probiotics are considered, the most defensible approach is to use products with fully disclosed strains and viable dose (CFU), and to align expectations with outcomes most plausibly supported by existing trials (symptom/QoL domains rather than consistent reductions in thyroid autoantibodies). In hypothyroidism (including patients treated with levothyroxine), a commonly studied synbiotic formulation has contained *Lactobacillus casei, L. acidophilus, L. rhamnosus, L. bulgaricus, Bifidobacterium breve, B. longum*, and *Streptococcus thermophilus* combined with fructo-oligosaccharides (total ~10^9^ CFU/g) administered for ~8–10 weeks; reported effects have primarily involved selected clinical outcomes with inconsistent effects on thyroid biochemistry or autoantibodies (58, 59). For Graves’ disease/Graves’ orbitopathy, interventional evidence is preliminary; one randomized study suggested possible effects on relapse-related outcomes after antithyroid therapy, but strain-level details and replication are needed before indication-specific guidance can be made (59, 60). Clinicians may consider reassessing thyroid function after a stable supplementation period (e.g., ~6–8 weeks) and interpreting any changes cautiously given concomitant diet/medication variability. Probiotics should be avoided or used only with specialist input in severely immunocompromised patients or those with critical illness. Overall, probiotics may be reasonable as a time-limited, symptom-oriented adjunct in selected patients, while emphasizing that high-quality, strain-specific RCTs in AITD are still lacking (10, 19, 56, 57).

#### 3.6.3 Dietary eliminations and the gluten-free diet

Interest in the gluten-free diet in HT stems from its association with celiac disease and hypotheses involving intestinal permeability and molecular mimicry (8, 17, 56). Meta-analyses do not support routine use of a gluten-free diet in patients without celiac disease (8, 56). A gluten-free diet significantly alters gut microbiota composition and may promote pro-inflammatory features. Unjustified use may increase the risk of nutritional deficiencies (6, 17, 61). Dietary elimination should therefore be individualized and guided by careful clinical assessment.

#### 3.6.4 Experimental strategies

Fecal microbiota transplantation and next-generation probiotics represent promising research avenues but remain at the preclinical or early clinical trial stage in AITD (8, 18, 21). Ongoing studies, such as IMITHOT, may provide high-quality evidence regarding the potential clinical applications of microbiota-targeted therapies (18).

## 4 Discussion

**Figure 3:**
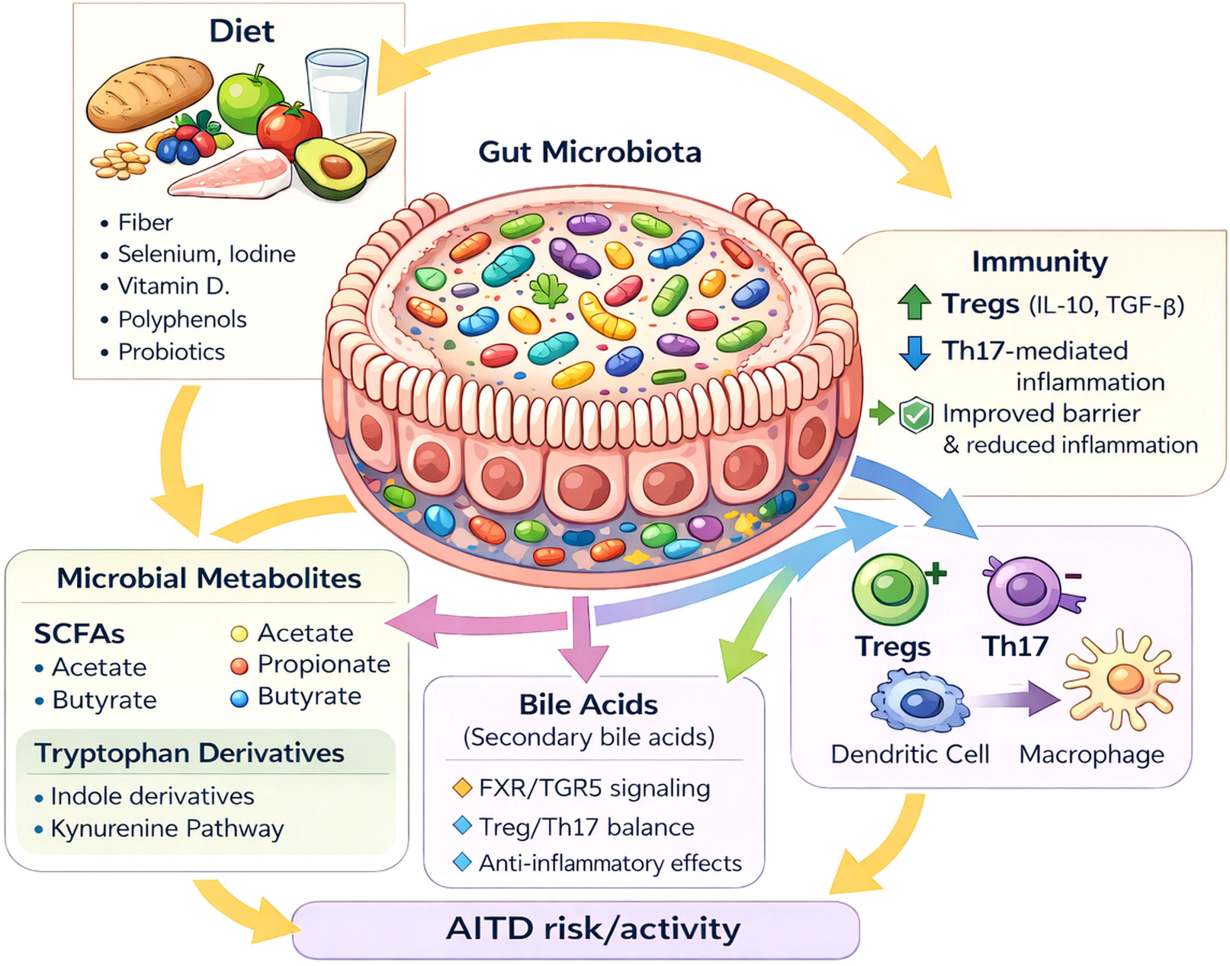
Diet–gut microbiota–metabolite–immune axis shaping autoimmune thyroid disease (AITD) risk and activity.

The variability in gut microbiota profiles reported in autoimmune thyroid diseases most likely reflects context-dependent functional shifts rather than a single, disease-specific taxonomic “signature.” The strongest signals across studies point to altered microbial function, including SCFA production, bile acid metabolism, and intestinal barrier integrity. These pathways integrate the effects of diet, micronutrient status (especially iodine), geography, and pharmacotherapy. They also shape immune mechanisms relevant to AITD, including Th17/Treg balance, antigen translocation, and potential molecular mimicry (6, 12, 14). A schematic overview of these diet–microbiota–metabolite–immunity pathways is shown in **Figure 3**. The current challenge is no longer to demonstrate that gut microbiota differs in autoimmune thyroid diseases, but to determine which functional alterations are clinically actionable—and in which patients.

**Figure 4:**
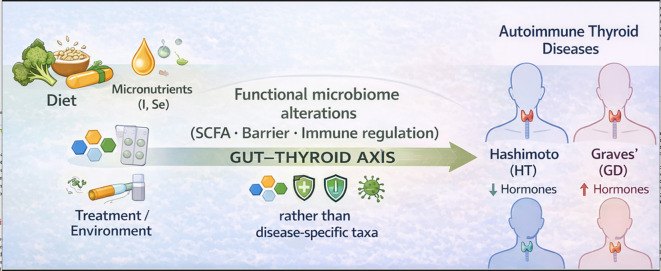
The gut–thyroid axis in autoimmune thyroid diseases.

Overall, available data support measurable differences in gut microbiota composition—and by extension, function—in both Hashimoto’s thyroiditis (HT) and Graves’ disease (GD) compared with healthy controls. Several studies also report associations between selected bacterial taxa and markers of thyroid autoimmunity, including anti-TPO, anti-Tg, and TRAb antibodies (6, 12, 14). These observations fit with the gut–thyroid axis as part of AITD pathophysiology. Still, translation to clinical practice requires caution, particularly when interpreting causality (**Figure 4**).

A major challenge is the lack of consistent direction of microbiota changes across studies. This likely reflects not only biological heterogeneity within AITD, but also overlapping clinical and environmental modifiers. Diet, iodine intake and status, supplementation practices, pharmacotherapy (levothyroxine, antithyroid drugs), and regional baseline microbiota patterns all contribute (6, 10–12). Geographic and dietary differences limit generalizability. Studies from different regions show marked heterogeneity, plausibly driven by distinct exposures and eating patterns (12, 15, 61, 62). For a focused discussion of how geographic and dietary factors confound cross-study comparisons and limit generalizability, see the ‘Geographic and dietary constraints’ subsection in the ‘Limitations of the Evidence’ section.

Taken together, it is increasingly unlikely that a universal microbiota profile exists for HT or GD. A more useful framework is to view observed differences as a set of functional, context-shaped alterations that may influence disease expression in predisposed individuals (6, 10, 11). In this setting, Mendelian randomization (MR) studies add value by suggesting potential causal links between specific microbial taxa and AITD. These findings remain hypothesis-generating and need validation in well-designed prospective clinical studies (13, 14).

### 4.1 Limitations of the evidence

Several recurring methodological and interpretive issues limit the current literature on gut microbiota in AITD. Predominance of cross-sectional studies. Most available data are cross-sectional. They identify associations but do not establish causality (6, 10–12). Prospective studies are scarce. As a result, it remains unclear whether dysbiosis precedes AITD or develops secondary to disease activity and/or treatment (6, 10, 11). MR can partly address temporality using genetic instruments, but MR results should be interpreted cautiously and tested in clinical cohorts (13, 14). As a narrative review, this work is inherently subject to selection bias; however, this limitation was mitigated by a structured search strategy, prioritization of higher-level evidence where available, and a critical appraisal framework emphasizing methodological quality and biological plausibility over isolated positive findings.

Geographic and dietary constraints. Study populations are often geographically homogeneous. This matters because the microbiota is strongly shaped by local diet, environmental exposures, and iodine status (12, 15, 61, 62). For example, microbiota shifts described in a Brazilian HT cohort (increased Bacteroides, reduced Bifidobacterium) may not replicate elsewhere (15). Dietary patterns with established anti-inflammatory and immunomodulatory effects, such as the Mediterranean diet, also modify microbiota composition and may influence AITD risk and disease course (61). Gut microbiota composition and function are strongly shaped by habitual diet and by region-specific exposures, including iodine fortification policies and iodine intake, micronutrient status, and broader environmental factors. Consequently, case–control contrasts observed in one geographic setting may reflect a local baseline microbiota configuration rather than a disease-specific pattern, limiting generalizability across populations. Cross-study comparisons are further confounded by differences in dietary assessment (or lack thereof), variability in fiber and ultra-processed food intake, fermented food consumption, and supplement use, all of which can shift microbial taxa and metabolites relevant to immune regulation. In addition, reverse causation is plausible: patients may change diet after diagnosis (e.g., adopting elimination diets or supplements), which can bias observational associations and inflate apparent “disease-related” microbiota differences. These issues likely contribute to inconsistent directionality of taxa-level findings across cohorts and support emphasizing convergent functional pathways over single taxonomic signatures. Future studies would benefit from standardized dietary assessment, systematic documentation of iodine exposure/status, and multi-center designs with harmonized protocols to improve comparability and external validity.

Geographic and methodological variability. Inter-study heterogeneity in gut microbial findings may partly reflect geographic and methodological variability. Geographic differences in diet, lifestyle, environmental exposures, socioeconomic factors, host genetics, and healthcare practices (including antibiotic and probiotic use) can shape baseline gut microbial communities and may modify observed associations with autoimmune thyroid disease phenotypes. In addition, differences in recruitment criteria and clinical characterization (e.g., disease stage, treatment status, iodine intake, comorbidities) may contribute to inconsistent signals across cohorts. Methodological factors further increase variability, including sample type and handling (stool vs. mucosal samples, storage temperature, freeze–thaw cycles), DNA extraction protocols, primer choice and targeted 16S rRNA regions, sequencing platforms, depth, and bioinformatic pipelines (quality filtering, taxonomic databases, normalization approaches). These sources of batch effects can alter relative abundances and diversity estimates, complicating cross-study comparisons. Future studies would benefit from harmonized protocols, transparent reporting of laboratory and analytic steps, and multi-center designs including diverse populations. Where feasible, standardized pipelines and meta-analytic approaches that account for batch effects and key covariates may improve reproducibility and help distinguish robust disease-related patterns from context-dependent variation.

Incomplete control of confounding. Many studies do not adequately account for BMI, smoking, antibiotic exposure, over-the-counter probiotic use, proton pump inhibitors, or gastrointestinal comorbidities such as celiac disease and IBS, all of which can substantially alter the microbiota (6, 10, 12, 63). Medication effects are another major issue. Commonly used drugs that influence the microbiota (e.g., levothyroxine, metformin, statins, PPIs) complicate interpretation in observational designs (12, 45).

Methodological heterogeneity in microbiota analysis. Differences in analytic approaches—16S rRNA sequencing versus shotgun metagenomics—targeting of different 16S regions, and non-uniform alpha and beta diversity metrics all reduce comparability across studies (6, 12, 64–66). Although 16S rRNA sequencing is widely available and practical for large cohorts, it offers only limited taxonomic granularity and, on its own, says little about what the microbiota is actually doing functionally (64, 66, 67). Shotgun metagenomics provides finer resolution and allows analysis at the pathway level, but the trade-off is higher cost and greater analytic complexity. In practice, shallow shotgun sequencing is often used as a compromise (16, 64–67).

Few interventional studies with clinically meaningful endpoints. Randomized trials with hard clinical outcomes are limited. Examples include changes in levothyroxine requirement, progression of thyroid dysfunction, GD relapse risk, or GO course (10, 11). Most trials focus on surrogate markers such as antibodies or inflammatory indices, often without long-term follow-up (10).

Separating cause from consequence. Distinguishing primary mechanisms from secondary effects is particularly difficult in AITD (6, 7, 10, 68, 69). Hypothyroidism slows intestinal transit, may reduce gastric acid secretion, and can predispose to SIBO. Hyperthyroidism accelerates transit and may cause diarrhea. These effects can reshape the microbiota independent of autoimmunity (68–70). Animal data show that induced thyroid dysfunction alters the microbiota, supporting a bidirectional gut–thyroid relationship (6, 71). Dysbiosis may therefore be part of a feedback loop rather than the initiating trigger (6, 7, 10).

Treatment effects on the microbiota. Levothyroxine and antithyroid drugs may themselves modulate the microbiota, further complicating cross-sectional comparisons (10, 12, 45, 71). Preclinical work suggests some changes may reverse after restoration of euthyroidism, but human clinical data remain limited (10, 71).

Finally, although circulating zonulin has been reported in AITD cohorts and is often discussed in the context of barrier function, its interpretation is limited by assay specificity (several commonly used commercial ELISAs may not reliably quantify pre-haptoglobin 2); therefore, zonulin should be viewed as a non-specific barrier-related signal and ideally interpreted alongside complementary markers (e.g., I-FABP, LPS/LBP/sCD14) or functional permeability testing when available.

Taken together, the data are biologically coherent and clinically interesting, but they are not yet practice-changing. What is needed now are well-designed prospective and interventional studies, with careful control of key confounders and more consistent methodology across cohorts (6, 7, 10, 11).

This review does not argue for microbiota-targeted interventions as disease-modifying therapy in autoimmune thyroid diseases, nor does it propose routine microbiota testing in clinical practice. Instead, it emphasizes careful interpretation of functional signals and their role in supportive, phenotype-informed care.

### 4.2 Clinical implications and practical approach

From a clinical standpoint, the most relevant implications involve interventions with established utility, guided by risk assessment and individualized care (**Table 2**).

**Table 2:** Evidence map of microbiome-relevant, nutrition- and gut-focused interventions in autoimmune thyroid diseases (AITD).

Intervention/exposure (what you do in clinic)	Primary functional domains targeted	AITD population/setting	Evidence base (most informative signal)	Clinical outcomes most consistently reported	Bottom-line clinical signal	Certainty of clinically meaningful benefit
Selenium 200 μg/day for 6 months	Immune profile; oxidative/inflammatory tone; autoantibodies (secondary)	Mild, active Graves’ orbitopathy (GO/TED)	Landmark RCT + guideline endorsement: improved course and QoL in mild GO; recommended in mild active GO in selenium-deficient areas	GO severity/progression; QoL; need for escalation	Use is reasonable and evidence-based in *mild active GO* (especially where selenium intake is low). Not a substitute for standard GO management; position as adjunct	High (for mild active GO)
Smoking cessation (risk-factor control, not “supplement”)	Key modifying factor; immune profile (indirect)	Graves’ disease with or without GO; highest priority in GO/TED	Strong, consistent guideline-level evidence: smoking may increase risk, severity, and post-RAI progression of GO; cessation urged for all GD patients	GO incidence/severity; progression after RAI; response to therapy	Non-negotiable adjunctive care: counsel actively, document, and support cessation; it is one of the few interventions with clear, reproducible impact on GO trajectory	High (risk modification)
Identify and treat GI malabsorption drivers (celiac disease, atrophic gastritis/H. pylori, hypochlorhydria)	Intestinal barrier/function (broad); key modifying factors (drug & micronutrient bioavailability)	Hypothyroidism on LT4 with unexpectedly high dose requirements or unstable TSH	Clinical reviews and cohort data: gastric disease and H. pylori/atrophic gastritis associated with higher LT4 dose requirement; celiac disease impairs absorption; improvement with treatment/diet in many cases	LT4 dose requirement; TSH stability; symptom control	High-yield clinical step: screen/treat when LT4 needs are disproportionately high or unstable. This is often more “practice-changing” than microbiome supplements	Moderate–High (for LT4 optimization)
Avoid high-dose iodine supplements (kelp/seaweed tablets, “thyroid support” blends)	Key modifying factor; immune activation (indirect)	Especially HT/AITD-prone individuals; also relevant in GD	ATA statement advises against iodine/kelp supplements >500 μg/day; excess iodine can trigger thyroid dysfunction and autoimmunity in susceptible patients	Thyroid dysfunction episodes; autoimmunity risk; disease destabilization	Counsel explicitly: iodine is essential, but *supplement megadoses are not benign* in AITD. Review OTC supplements and “natural thyroid” products	Moderate (harm avoidance)
Vitamin D supplementation (when deficient/insufficient)	Immune profile (Th17/Treg axis—indirect); barrier (secondary)	Mainly HT (and broader AITD)	Meta-analyses of RCTs: signals for reductions in thyroid antibody titres in some analyses; thyroid function outcomes inconsistent	Anti-TPO/anti-Tg; TSH/fT4; inflammatory markers	Treat deficiency for general health; do not promise endocrine “remission.” Reasonable adjunct if low 25(OH)D, but set expectations: antibodies may fall; clinical impact often unclear	Low–Moderate
Probiotics/prebiotics (selected use, not routine)	SCFA/metabolic output; barrier; immune profile	AITD with GI symptoms; some interest in GD (TRAb)	Recent meta-analysis of RCTs: no meaningful change in TSH/fT3/fT4 overall; possible modest TRAb reduction in GD; high heterogeneity (strain/dose/duration)	TRAb (GD); QoL; GI symptoms; thyroid hormones	Consider selectively (GI symptoms, antibiotic-associated dysbiosis-like symptoms, patient preference) and be explicit that endocrine effects are typically small/absent; avoid “one-size-fits-all” claims	Low
Synbiotics (pro+pre) as adjunct	SCFA output; barrier; immune profile	Mixed thyroid cohorts; limited AITD-specific RCT data	Evidence is sparse and heterogeneous; difficult to generalize across formulations	TSH/fT3/fT4; inflammatory indices; QoL	Not routine. If used, treat as symptom-directed adjunct with careful strain/formulation choice; monitor expectations and stop if no benefit	Very low–Low (limited, inconsistent)
Gluten-free diet in non-celiac HT	Barrier/immune hypotheses (indirect); microbial diversity shifts	HT without diagnosed celiac disease	Systematic review/meta-analysis (non-celiac HT): no consistent benefit on thyroid hormones; mixed antibody effects; evidence rated very uncertain with major methodological limitations	TSH/fT3/fT4; anti-TPO/anti-Tg	Do not recommend routinely. Reserve for confirmed celiac disease, strong clinical suspicion awaiting workup, or clear patient-specific indications; avoid nutritional harm from unnecessary restriction	Very low
Diet quality upgrade (Mediterranean-style pattern; fiber-first; reduce ultra-processed foods)	SCFA output; barrier integrity; immune tone; microbial diversity	All AITD (adjunct to standard therapy)	Mostly indirect + observational + mechanistic coherence (strongest at functional level: SCFA/barrier) rather than AITD-specific RCT endpoints	Inflammatory indices; GI symptoms; cardiometabolic risk; QoL; occasionally antibodies	Best “low-risk, high-plausibility” baseline. Frame as foundational lifestyle medicine (gut function, cardiometabolic health), not as a replacement for LT4/ATD/definitive GD therapy	Low (AITD endpoints), Moderate (overall health)
Correct iron deficiency (and other confirmed micronutrient deficits: zinc, B12 when indicated)	Key modifying factor (thyroid physiology + drug response); immune tone (secondary)	AITD with documented deficiency, malabsorption risk, heavy menses, etc.	Clinical physiology + practice-level evidence: deficiencies are common and clinically relevant; improves general symptoms and can affect thyroid management (e.g., response to LT4)	Symptoms, ferritin/TSAT; TSH stability (selected patients)	Do it—because it’s good medicine. This is standard-of-care optimization; microbiome framing is secondary	Moderate (for patient-centered benefit in deficiency)

AITD, autoimmune thyroid diseases; HT, Hashimoto’s thyroiditis; GD, Graves’ disease; GO/TED, Graves’ orbitopathy/thyroid eye disease; LT4, levothyroxine; ATD, antithyroid drug; TRAb, TSH-receptor antibodies; QoL, quality of life; SCFA, short-chain fatty acids.

First, clinicians should identify and treat comorbid gastrointestinal disorders, especially celiac disease, which can impair drug and micronutrient absorption. Elimination diets should be implemented primarily when clear indications are present (8, 21, 69). Second, iodine intake and status should be assessed regularly. Both deficiency and excess may exacerbate autoimmunity. Particular caution is warranted in patients using iodine supplements or consuming iodine-rich diets (8, 54).

Next, micronutrient deficiencies should be identified and corrected, including selenium, iron, zinc, and vitamin D. These deficits are common in AITD and may worsen disease expression. Supplementation should be guided by nutritional assessment and laboratory testing rather than routine use (19, 20, 54). Dietary counseling should emphasize a pattern that supports microbiota homeostasis: higher fiber intake (vegetables, fruit, whole grains), moderate use of fermented foods, and reduced intake of ultra-processed foods and saturated fats. A Mediterranean or broadly anti-inflammatory dietary pattern is a reasonable template (8, 19, 72, 73).

Probiotics and synbiotics may be considered in patients with gastrointestinal symptoms or in selected clinical settings. Current data suggest modest benefits, mainly for quality of life and potentially for TRAb reduction in GD, with little effect on thyroid hormone parameters. Routine use in all patients cannot be recommended at present (17, 18, 21). Notably, the same principles that guide pragmatic adjunctive care today should also inform the design of future microbiota-focused studies. For clarity, the key practical implications of the available evidence for clinicians are summarized in **Box 1**.

Box 1 **Clinical pragmatics: how to use gut- and nutrition-oriented adjuncts in AITD**

These measures are adjuncts. They can improve treatment stability, symptom burden, and comorbidity management, but they do not replace standard thyroid care (LT4 titration in hypothyroidism; antithyroid drugs/RAI/surgery in Graves’ disease; guideline-based management of GO/TED).

Step 1 — Get the basics right (this solves more than most supplements)

• Confirm phenotype: Hashimoto’s (HT) vs Graves’ (GD); document GO/TED presence and activity.

• Review levothyroxine (LT4) administration carefully: fasting intake, consistent timing, separation from iron/calcium, coffee, bile-acid binders; check adherence and formulation switches.

• If TSH is “unstable,” assume an absorption or interaction issue until proven otherwise.

Step 2 — When to look for GI contributors (and what to test)

Think “GI-driven instability” when you see:

• Unexpectedly high LT4 requirements, recurrent dose escalations, or fluctuating TSH despite reasonable dosing and reported adherence.

• Iron deficiency, B12 deficiency, chronic GI symptoms, weight loss, or broader autoimmune clustering.

Practical work-up (tailor to presentation):

• Celiac disease: tTG-IgA plus total IgA (use IgG-based assays if IgA deficient).

• H. pylori/atrophic gastritis: consider testing when dyspepsia, iron deficiency, B12 deficiency, or long-term acid suppression is present; treat if positive/indicated.

• When malabsorption is plausible, address the cause first; consider LT4 formulation adjustments (e.g., liquid/soft-gel) only as part of a broader plan.

Step 3 — Micronutrients: correct deficiencies; avoid “blanket” supplementation

• Iron: check ferritin/TSAT when fatigue is prominent, menses are heavy, diet is restrictive, or LT4 stability is poor; replete if deficient (and separate dosing from LT4).

• Vitamin D: correct deficiency for general health; set expectations—this is not a disease-modifying therapy for AITD.

• Selenium: the clearest clinical niche is mild, active GO/TED as an adjunct (particularly where selenium intake is low). Avoid prolonged high-dose use; be mindful of total intake from diet and supplements.

• Iodine: actively ask about OTC “thyroid support” products, kelp, and high-iodine supplements. In AITD-prone patients, avoid high-dose iodine unless there is a clear medical indication.

Step 4 — Diet: improve quality first; restrict only for a reason

• Default advice: a Mediterranean-style, minimally processed, fiber-forward pattern with adequate protein and micronutrient density.

• Gluten-free diet:

• Indicated in confirmed celiac disease.

• Not a routine recommendation in non-celiac HT; the evidence for thyroid outcomes is inconsistent and the risk of nutritional compromise is real.

• If patients pursue restrictive diets anyway, involve a dietitian to prevent deficiencies and maintain long-term adherence safely.

Step 5 — Probiotics/synbiotics: a selective, symptom-driven trial—not endocrine therapy

Reasonable to consider when:

• GI symptoms are clinically relevant (bloating, altered bowel habits), especially after antibiotics or during diet transitions.

• The patient wants a low-risk adjunct and understands the goal.

How to position it:

• Aim for GI symptom support, not normalization of TSH/FT4 or “turning off” antibodies.

• Use a time-limited trial and stop if there is no benefit.

• Avoid in patients with severe immunosuppression or where safety is uncertain; choose strain-identified, reputable products.

Step 6 — Graves’ disease and GO/TED: don’t miss the interventions that actually change risk

• Smoking cessation should be addressed explicitly and repeatedly; it is one of the few modifiable factors consistently linked to GO/TED risk and severity.

• Coordinate GO/TED management with ophthalmology; adjunct nutrition/microbiome measures should not delay proven therapies.

Step 7 — Monitoring: keep it clinically anchored

• Recheck thyroid function after any change likely to affect absorption or dosing stability (iron therapy, GI treatment, major dietary restriction), using standard reassessment intervals.

• Use antibodies as secondary context; prioritize symptoms, biochemical control, dose stability, and phenotype-specific outcomes (especially in GD/GO).

### 4.3 Future research priorities

Rather than expanding descriptive microbiota catalogues, future progress in this field will depend on more focused and clinically oriented study designs. From a practical perspective, four elements are likely to be critical for generating clinically actionable evidence:

- Who: clearly phenotyped patient populations, with systematic documentation of iodine status, disease stage, treatment status (euthyroid vs treated), and relevant gastrointestinal comorbidities.
- What: function-focused readouts, prioritizing microbial metabolites and pathways (e.g., SCFA profiles, bile acid metabolism) and validated markers of intestinal barrier integrity rather than taxonomic shifts alone.
- How: integrated multi-omics approaches combining metagenomics with metabolomics and targeted immune profiling, allowing functional signals to be linked to immunological and endocrine phenotypes.
- Endpoints: clinically meaningful outcomes, such as levothyroxine dose requirements and stability, relapse risk in Graves’ disease, or progression and activity of Graves’ orbitopathy, rather than antibody titers alone.
- Suggested study designs to improve causal inference and clinical translation: To move beyond descriptive catalogues, the field would benefit from a small number of rigorously designed, hypothesis-driven study formats: (1) Prospective, multi-center longitudinal cohorts with repeated sampling (baseline, early follow-up, and post-treatment timepoints) integrating shotgun metagenomics with metabolomics (SCFA/bile acids), targeted immune profiling, and standardized dietary and iodine exposure assessment. Such cohorts should predefine hard clinical endpoints (e.g., levothyroxine dose requirement and TSH stability over time; progression from subclinical to overt hypothyroidism; relapse after antithyroid drug withdrawal in Graves’ disease; and activity/severity trajectory in Graves’ orbitopathy); (2) Inception cohorts of newly diagnosed, treatment-naïve patients, ideally enriched for early disease, can strengthen temporality and reduce reverse causation by capturing microbiota–immune signals before major dietary changes or pharmacotherapy effects; (3) Pragmatic randomized designs, including factorial trials (e.g., diet-quality intervention × microbiota-targeted adjunct such as a standardized synbiotic/postbiotic), or adaptive platform trials for multiple low-risk interventions, would allow efficient testing against clinically meaningful outcomes with adequate follow-up. Across designs, harmonized protocols (sample collection, sequencing pipelines, diet/iodine measures, medication documentation) and replication across regions are critical to improve comparability and external validity. Autoantibody titers may be retained as secondary outcomes, but primary endpoints should prioritize clinically meaningful measures and longer follow-up.

Promising strategies include microbiota- and metabolite-informed personalized nutrition, bacterial consortia, and postbiotics. Their efficacy and safety still require confirmation in clinical trials (6, 10, 62). Future work should move beyond searching for single taxonomic “signatures” and instead stratify patients by microbial function and test interventions against measurable, clinically relevant endpoints.

Shifting the focus from descriptive differences toward functional, phenotype-informed, and outcome-oriented study designs will be essential to translate microbiota research into clinical relevance in autoimmune thyroid diseases.

## 5 Conclusions

Available evidence supports a bidirectional gut–thyroid axis in which the gut microbiota and its metabolites influence intestinal barrier function, immune regulation, and micronutrient homeostasis relevant to thyroid physiology. At present, the gut microbiota should be viewed as a modifier of disease expression rather than a primary driver of autoimmune thyroid diseases. Dysbiosis is reported in AITD, but no uniform and reproducible microbiota profile has been identified. Findings should be interpreted in light of diet, geographic variation, and treatment effects.

In clinical practice, the most defensible and safest strategy remains dietary optimization, with particular emphasis on increasing dietary fiber and improving overall diet quality. Probiotics, prebiotics, and synbiotics may be considered selectively as adjunctive measures, but current data do not support their routine use in AITD.

Regardless of any potential microbiota modulation, clinical priorities include identifying and correcting micronutrient deficiencies, avoiding iodine excess, and diagnosing and treating comorbid gastrointestinal disorders. Prospective and randomized studies are needed to define the true clinical value of microbiota-targeted interventions in autoimmune thyroid diseases.

In routine practice, the sensible priorities remain the basics: overall diet quality, a realistic appraisal of iodine exposure, and structured evaluation of micronutrient status and relevant comorbidities. Microbiota-directed approaches, where considered, should be positioned as adjuncts for selected patients—never as a replacement for established, evidence-based therapy.

In this context, microbiota-targeted interventions cannot currently be recommended as routine care; instead, improving diet quality, addressing modifiable nutritional risks, and managing gastrointestinal comorbidities represent the most evidence-aligned ways to engage the gut–thyroid axis in routine clinical practice.
